# Role of cMET in the Development and Progression of Colorectal Cancer

**DOI:** 10.3390/ijms140918056

**Published:** 2013-09-03

**Authors:** Juan Carlos Samamé Pérez-Vargas, Pamela Biondani, Claudia Maggi, Manuela Gariboldi, Annunziata Gloghini, Alessandro Inno, Chiara Costanza Volpi, Ambra Vittoria Gualeni, Maria di Bartolomeo, Filippo de Braud, Alessandra Castano, Ilaria Bossi, Filippo Pietrantonio

**Affiliations:** 1 Medical Oncology Department, Arnau de Vilanova Universitary Hospital, 25198 Lleida, Spain; E-Mail: jcspv@hotmail.com; 2 Medical Oncology Department, Fondazione IRCCS Istituto Nazionale dei Tumori, Via Venezian, 1-20133 Milan, Italy; E-Mails: pamela.biondani@istitutotumori.mi.it (P.B.); claudia.maggi@istitutotumori.mi.it (C.M.); maria.dibartolomeo@istitutotumori.mi.it (M.B.); filippo.debraud@istitutotumori.mi.it (F.B.); alessandra.castano@istitutotumori.mi.it (A.C.); ilaria.bossi@istitutotumori.mi.it (I.B.); 3 Experimental Oncology and Molecular Medicine Department, Fondazione IRCCS Istituto Nazionale dei Tumori, Via Venezian, 1-20133 Milan, Italy; E-Mail: manuela.gariboldi@istitutotumori.mi.it; 4 FIRC Institute of Molecolar Oncology Foundation (IFOM), 1-20133 Milan, Italy; 5 Pathology Department, Fondazione IRCCS Istituto Nazionale dei Tumori, Via Venezian, 1-20133 Milan, Italy; E-Mails: annuziata.gloghini@istitutotumori.mi.it (A.G.); chiara.volpi@istitutotumori.mi.it (C.C.V.); ambra.gualeni@istitutotumori.mi.it (A.V.G.); 6 Medical Oncology, Sacro Cuore-Don Calabria Hospital, 37024 Negrar (Verona), Italy; E-Mail: alessandro.inno@gmail.com

**Keywords:** colorectal cancer, hepatocyte growth factor, mesenchymal-epithelial transition factor, pathogenesis, prognosis

## Abstract

Mesenchymal-epithelial transition **(***MET*) is a member of a distinct subfamily of heterodimeric receptor tyrosine kinase receptors that specifically binds the hepatocyte growth factor (*HGF*). Binding to *HGF* leads to receptor dimerization/multimerization and phosphorylation, resulting in its catalytic activation. *MET* activation drives the malignant progression of several tumor types, including colorectal cancer (CRC), by promoting signaling cascades that mainly result in alterations of cell motility, survival, and proliferation. *MET* is aberrantly activated in many human cancers through various mechanisms, including point mutations, gene amplification, transcriptional up-regulation, or ligand autocrine loops. *MET* promotes cell scattering, invasion, and protection from apoptosis, thereby acting as an adjuvant pro-metastatic gene for many tumor types. In CRC, *MET* expression confers more aggressiveness and worse clinical prognosis. With all of this rationale, inhibitors that target the HGF/*MET* axis with different types of response have been developed. *HGF* and *MET* are new promising targets to understand the pathogenesis of CRC and for the development of new, targeted therapies.

## 1. Introduction

Colorectal cancer (CRC) is the second leading cause of cancer-related deaths worldwide, and represents the most frequent gastrointestinal malignancy in Western countries [1]. Despite the advances in the management of CRC, about 30% of patients eventually develop distant metastases after curative surgery, even when treated with adjuvant chemotherapy and/or radiotherapy. The molecular biology of CRC has been widely studied [2]; however, the validation of biomarkers, which may help to predict regional and distant invasion, is still an unmet clinical need.

The mesenchymal-epithelial transition (*MET*) gene was discovered in 1984 in a human cell line of osteogenic sarcoma treated with the chemical carcinogen *N*-methyl-*N*-nitro-*N*-nitrosoguanidine [3]. *MET* oncogene is activated by the translocated promoter region (TPR), which translocates from chromosome 1 to the region upstream of the *MET* gene, on chromosome 7. The resulting TPR-*MET* fusion protein shows constitutively-active *MET* kinase activity [3,4].

During the same decade, a potent mitogen for parenchymal liver cells, the hepatocyte growth factor (HGF), was isolated in human plasma and murine platelets [5]. In addition, Stoker *et al.* [6] described the fibroblast-derived epithelial motility factor, or scatter factor (SF), a protein expressed by fibroblasts and smooth muscle cells that induces motility of epithelial cells [3]. Subsequent studies identified HGF and SF as the same protein (HGF/SF) [7]. Noteworthy, the *MET* proto-oncogene encodes for cMET, a receptor with tyrosine-kinase activity the only known ligand for which is HGF [8,9].

The cMET-HGF/SF pathway plays a crucial role in several biological activities such as motility, proliferation, cell survival, embryogenesis, angiogenesis, and wound healing [10–12]. However, this pathway is also involved in the development and metastatic progression of many different tumor types, including CRC and gastric cancer, ovarian cancer, head and neck squamous cell carcinoma, lung cancer, and hereditary and sporadic papillary renal cancer [13–18].

This review provides an update of the most significant preclinical and clinical data on the role of cMET in the development of CRC, exploring its possible use as prognostic biomarker and its potential applications as a predictive factor for pharmacological interventions.

## 2. Literature Search Methodology

For this review, the PubMed database was searched for articles concerning cMET as a biomarker for CRC and published in English before April 2013; early-release publications were also considered for inclusion. We used the search terms “colorectal cancer” AND “MET”. Preclinical and clinical studies were eligible if they evaluated the association of cMET with pathogenesis, pathological features, prognosis, or prediction of treatment outcomes in CRC, according to Authors’ judgment.

## 3. Characteristics of *MET* and Its Role in CRC

### 3.1. Molecular Biology of HGF/cMET Axis

**Figure 1:**
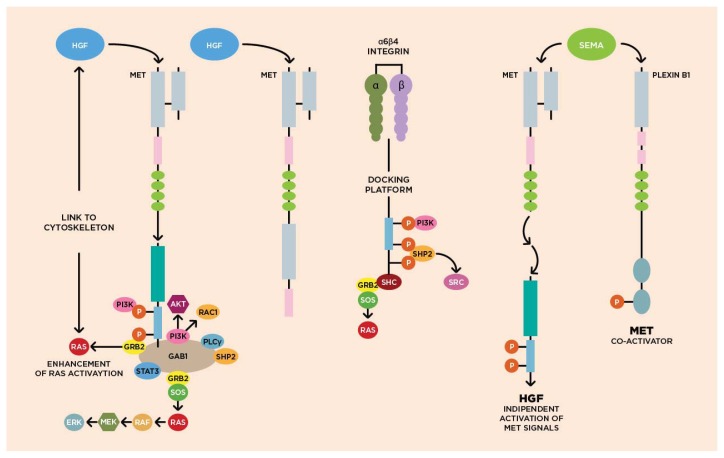
HGF/cMET signaling pathways. HGF–SF binds to and results in dimerization and activation of the c-Met kinase domain. The Tyr residues in the docking sites are then phosphorylated. Effector molecules such as GRB2, GAB1, SHP2, SOS, PLC, and SRC are recruited and activate a variety of downstream signaling cascades, chiefly the ERK–MAPK and the PI3K–AKT pathways.

The *MET* gene is located on chromosome 7 (bands q21–q31) and consists of 21 exons separated by 20 introns [19,20]. The extracellular domain of cMET presents two subunits, linked by a disulphide bond, which form the mature receptor for HGF. The intracellular domain is constituted of a juxtamembrane domain, involved in the receptor down-regulation, a tyrosine kinase domain, involved in signal transduction, and a *C*-terminal regulatory tail (Figure 1) [9,21].

cMET belongs to a heterodimeric receptor tyrosine kinase family, which includes the macrophage stimulating 1 receptor and has homology with semaphorins and plexins [22]. The cMET-related tyrosine kinase family also shares homology with the human insulin receptor [9]. cMET is physiologically expressed in epithelial cells, but is also found in vascular and lymphatic endothelial cells [23,24], as well as neural cells, hepatocytes, hematopoietic cells, and perycites [25–28].

HGF belongs to the plasminogen-related growth factor family. The *HGF* gene is composed of 70,000 base pairs (18 exons and 17 introns) and is located on chromosome 7q21.1. HGF protein belongs to the plasminogen-related growth factor family and it is expressed by cells of mesenchymal origin or by tumor cells through autocrine mechanism [10,29].

As shown in Figure 1, the activation of HGF/cMET pathway begins with the autophosphorylation of tyrosine residues of the intracellular region of cMET (Y1230, Y1234, Y1235) [30]. Further autophosphorylations on Y1349 and Y1356, two tyrosine residues near the COOH tail, form a multifunctional docking site that recruits intracellular adapters via SRC homology-2 domains and other recognition motifs, thus, initiating downstream signaling. Several proteins and kinase substrates, such as growth factor receptor-bound protein 1 (GRB1) and 2 (GRB2), phosphatidylinositol 3-kinase (PI3K), and v-src sarcoma viral oncogene homolog (SRC), act as adaptors [31,32]. In details, GRB1 tyrosyl phosphorylation by the cMET tyrosine kinase leads to the recruitment of PI3K, which in turn binds to cMET through its p85 subunit, and contributes to cell cycle progression, inhibition of apoptosis, and cellular motility [33].

### 3.2. Biological Activity of HGF/cMET Axis

The HGF/cMET pathway is related to many cellular and biological processes, as summarized in Table 1.

#### Embryogenesis

*MET* contributes to the migration and development of muscle tissue by controlling the epithelial-mesenchymal transition (EMT) of myogenic progenitor cells, and to the development of neuronal precursors, liver, and placental tissue. In fact, an animal study in mice knocked-out for either *HGF* or *MET*, or both, resulted in embryonic lethality [34].

#### Tissue regeneration

*MET* and *HGF* genes were reported to be up-regulated after injury in different epithelial tissues, such as kidney, lung, skeletal muscle, heart, skin, and liver. In the skin, *MET* was shown to be essential for wound repair [27]. In the liver, it was observed that the activation of the HGF/cMET pathway is essential for DNA synthesis and liver regeneration [35], while, on the other hand, *MET* ablation resulted in impaired proliferation and incomplete liver regeneration [36]. This suggests a role for *MET* in the protection against tissue damage and in tissue repair [12,18,31].

#### Cell proliferation and survival

HGF/cMET pathway was shown to provide tumor cells with a proliferative advantage, through the tyrosine phosphorylation of the focal adhesion kinase (FAK) and the downstream activation of cell proliferation, survival, and migration [37].

#### Cytoskeleton

HGF/cMET pathway induces tyrosine phosphorylation of paxillin, a protein involved in cell adhesion, actin reorganization, and cell growth [3,30].

#### Scattering and cell motility

HGF/cMET pathway increases cell motility, invasion, and, ultimately, metastases by acting on the cytoskeleton. PI3K is an important molecule in HGF-induced mitogenesis, morphogenesis, and chemotaxis [3,10,30,38,39].

### 3.3. cMET Signaling Pathway and Angiogenesis

The HGF/cMET signaling pathway plays a role in angiogenesis and lymphangiogenesis by promoting the growth of endothelial cells, increasing the expression of pro-angiogenic mediators, such as vascular endothelial growth factor (VEGF), and suppressing the activity of thrombospondin 1—a negative regulator of angiogenesis [23,40].

cMET activates several pathways including SRC/FAK, the signal transducer and activator of transcription 3 (STAT3), PI3K/AKT, and RAS [3,10]. These signaling pathways may stimulate endothelial cells both directly—by mitogenic or morphogenic activity—and indirectly by regulating other pro-angiogenic factors [40].

Hypoxia is a key regulator of cMET, as it induces the expression of the transcription factor hypoxia inducible factor 1 alfa (HIF-1α). The existence of this correlation is supported by preclinical studies in mouse xenograft models, which showed that the therapeutic inhibition of angiogenesis reduces tumor vascularization and causes hypoxia, and therefore may promote cMET-mediated invasion of malignant cells [41,42].

Considering that the relevant role of *MET* in angiogenesis is combined—in a synergic fashion—with the effect exerted by the VEGF/VEGFR pathway, novel therapeutic strategies, which focus on the simultaneous blockade of both pathways, have been recently proposed [43].

### 3.4. cMET and Other Growth Factor Receptors

The HGF/cMET axis presents several cross-talks with other growth factor receptors, such as the epidermal growth factor receptor (EGFR), the insulin growth factor receptor 1 (IGF1R), and the recepteur d’origine Nantais (RON). These cross-talks may help the understanding of the mechanisms of resistance to targeted therapies [44].

Specifically, the cross-talk between cMET and epidermal growth factor receptor (EGFR) is implicated in tumorigenesis. Jo *et al.* [45] reported that cMET is activated directly by the transforming growth factor alfa (TGFα) and EGFR in an autocrine fashion; however, indirect activation is also possibly induced by EGFR blockade. *MET* signaling also activates cells resistant to EGFR tyrosine kinase inhibitors. In non-small-cell lung cancer (NSCLC), Engelman *et al.* [46] showed that cMET amplification could explain the resistance to EGFR tyrosine kinase inhibitors, such as gefitinib. Liska *et al.* [47] showed that EGFR and cMET are co-expressed in CRC cell lines, and act synergistically to increase proliferation. It is well known that therapeutic strategies for CRC have focused on the development of anti-EGFR monoclonal antibodies (MoAbs)—such as cetuximab and panitumumab [48,49]. The emergence of resistance to cetuximab was explained by HGF-mediated activation of cMET, with increased signaling through the PI3K/AKT and mitogen-activated protein kinase (MAPK) pathways. Therefore, dual blockade of cMET and EGFR may be synergistic in the treatment of CRC [47].

The HGF/cMET axis presents a well-established cross-talk with IGF1 and its receptor IGF1R, which are both implicated in the development and progression of a variety of human cancers. Bauer *et al.* [50] demonstrated that IGF1R and cMET contribute synergistically in human CRC cells to the activation of the urokinase plasminogen activator and its receptor, which are mediators of migration and invasion. In a recent study, Varkaris *et al.* [51] proved that IGF1 expression is sufficient to induce *MET* activation *in vivo* in a xenograft model. In multiple cell lines, IGF1-mediated *MET* activation suggests that this cross-talk may contribute to progression in several cancer types when both molecules are expressed. This finding suggests that *MET* may be activated by multiple tyrosine kinase receptors, and may therefore be an important therapeutic target [51].

RON is a member of the *MET* tyrosine kinase receptor family, which is associated with resistance to apoptosis, production of superoxide anions, and phagocytosis of macrophages. Therefore, it stimulates pro-oncogenic signaling pathways such as SRC and PI3K/AKT [52]. The cross-talk between cMET and RON was documented in a number of experimental models, and was confirmed in human cancers including liver, pancreas, breast, and bladder carcinomas [53]. In hepatocellular carcinoma, some cytokines, including epidermal growth factor (EGF), interleukin1, interleukin6, and TNFα, are able to induce the expression of both cMET and RON, thus suggesting that *MET* and RON are regulated by similar cytokine networks [54]. RON/cMET heterodimerization plays a key role in the activation of related signal transduction pathways. Follenzi *et al.* [54] showed that the formation of the heterodimeric complexes leads to reciprocal transphosphorylation on tyrosine residues of the two receptors, therefore resulting in the activation of their catalytic regions. In particular, not only is RON specifically phosphorylated by an activated form of cMET, but the presence of *MET*-specific inhibitors suppresses RON phosphorylation.

### 3.5. MET Mutation and Deregulation

Aberrant HGF/cMET signaling pathway was described in several solid tumors. The mutation of *MET*, located in the tyrosin-kinase domain, was first described in type 1 hereditary papillary renal carcinoma and in sporadic papillary renal carcinoma [55]. *MET* mutations may also, rarely, be oncogenic drivers in metastatic head and neck cancer [15], gastric cancer [16], liver cancer [36], and NSCLC [17]. Other mutations can also be found in different regions of cMET, such as the iuxtamembrane region, and result in the receptor’s up-regulation—as was shown in 12% of NSCLC [17,31].

*MET* mutations, overexpression of HGF or cMET, and co-expression of both HGF/SF and *MET* by the same cell, can all contribute to tumorigenesis. In fact, cell lines overexpressing cMET present a highly invasive and metastatic potential [37], due to the up-regulation of urokinase-type plasminogen activator and matrix metalloproteinases [56]. Other mechanisms include the induction of angiogenesis, lymphangiogenesis, and the prevention of apoptosis through the phosphorylation of PI3K and subsequent AKT activation [30,57]. HGF overexpression appears to be related to the invasion and migration of tumor cells, mainly through the p42/p44 MAPK pathway, which enhances cellular proliferation, promoting the EMT [58]. *In vitro* studies showed that the induction of increased HGF levels stimulates the invasiveness of Caco-2 CRC, by promoting cell motility and proteases synthesis [59].

## 4. Methods of cMET Assessment

The oncogene *MET* can be studied both at the protein and gene levels. In order to investigate the protein expression, immunohistochemistry (IHC) represents a reliable technique, and a large number of antibodies effective in formalin fixed paraffin embedded (FFPE) tissues and able to recognize different domains, either in native, or phosphorylated forms of the receptor are commercially available. The selection of the antibody should be based on the specific aim of the investigation: some antibodies recognize residues near the *N*-terminus of cMET, while others recognize residues near the *C*-terminus. For instance, if cMET detection is used to identify patients eligible for treatment with a monoclonal antibody, which blocks the receptor activity by targeting the extracellular domain of cMET, then an antibody directed against residues near the *N*-terminus should be used. Moreover, some antibodies match the non-phosphorylated domain of the protein, while others recognize only the phosphorylated form. The former are useful to measure the amount of mature protein, while the latter are useful to evaluate the activated form.

A consensus on the evaluation criteria of the IHC results has not been reached yet. Literature data are scant, and, usually, criteria used for the assessment of other biomarkers (e.g., estrogen receptors, HER2, EGFR) in breast, gastric, or lung carcinoma, are extended to the evaluation of cMET expression in CRC. A semi-quantitative assessment—the *H*-score—has been described [60–62]. The original concept of the *H*-score [60] combines staining intensity (scored from 0 to 4) with the percentage of positive cells (scored 0%–100%). Each single intensity level is multiplied by the percentage of cells, and all values are summed up to obtain the final *IHC* score, which ranges from 0 to 400. Scores from 0 to 200 are considered to be associated with negative/low expression, while scores from 201 to 400 are considered to show positive/high expression [60–62]. A modified *H*-score system has also been developed [63]. In this scoring system, three staining intensity levels (scored from 0 to 3) are considered. This modified *H*-scoring system provides a total score ranging from 0 to 300; cases are classified as negative (score 0–50), weakly positive (51–100), moderately positive (101–200), or strongly positive (201–300) [63]. Another method used to evaluate the expression of cMET is similar to that applied to HER2. More specifically, samples are classified as negative (0, 1+), when no staining or faint staining is present in <10% of cells; ambiguous (2+) when moderate staining is present in >10% of cells; positive (3+), when a circumferential, basolateral, or lateral signal for cMET over-expression of protein with strong intensity is present in >10% of the cells [64].

Although IHC is the most commonly-used method to examine cMET expression, it cannot establish whether the receptor over-expression is actually due to gene amplification or to other mechanisms, such as transcriptional activation or hypoxia [65]. To assess the amplification of *MET*, *in situ* hybridization techniques should be performed. Both fluorescence *in situ* hybridization (FISH) and single or double silver *in situ* hybridization (SISH) enable the measurement of the number of gene copies and of the chromosome 7 centromere copy number. *MET* amplification can be defined according to what established for *HER2* testing [66], in which amplification is defined as a gene-to-centromere ratio (*MET*/CEP7) ≥2.2 or *MET* copy number ≥6. Alternatively, the method described for EGFR [61] is still used to evaluate the *MET* status [67]. Amplification of *MET* is classified into six groups as follows: (i) disomy (≤2 copies in ≥90% of cells); (ii) low trisomy (≤2 copies in ≥40% of cells, 3 copies in 10%–40% of cells, ≥4 copies in <10% of cells); (iii) high trisomy (≤2 copies in ≥40% of cells, 3 copies in ≥40% of cells, ≥4 copies in <10% of cells); (iv) low polysomy (≥4 copies in 10%–40% of cells); (v) high polysomy (≥4 copies in ≥40% of cells); and (vi) gene amplification (defined by the presence of tight *MET* clusters and a ratio of *MET*/CEP7 ≥2, or ≥15 copies of *MET*/cell in ≥10% of analyzed cells). High polysomy and gene amplification are considered as a positive SISH result, while the others represent negative results.

## 5*. MET* and the Pathogenesis of Colorectal Cancer

The role of *MET* in the pathogenesis of CRC has been extensively described [68–72]. The measurement of the expression of *MET* mRNA and related cMET protein in CRC can vary according to the technique used and to the number of samples analyzed. Results range between 30% and 91% with Northern blot and polymerase chain reaction (PCR) assays, and between 57% and 100% by Western blot and IHC analyses [73–75].

Most of the reports are consistent in showing that cMET expression is higher in the metastases than in the primary tumor tissue. Zeng *et al.* [72] compared *MET* gene copies in normal tissues, primary CRC, and liver metastases, by using highly quantitative PCR/ligase detection reaction technique. No differences between normal colonic mucosa and liver parenchyma were observed; however an increase in *MET* expression was reported in primary CRC compared with normal mucosa, and in liver metastases compared with normal liver tissue. Interestingly, a significant increase in cMET copies was observed in liver metastases compared with primary CRC tumors (18% *vs.* 2%, *p* < 0.001), suggesting that the amplification of this gene is a late event in CRC progression and is associated with hematogenous dissemination. Therefore, while *MET* amplification may be a rare event in localized CRC, it is more common in advanced tumor stages [72].

Di Renzo *et al.* [73] studied the changes of *MET* gene expression during the progression of CRC from adenoma to metastatic cancer. Overexpression was detectable in about 50% of tumors, at any stage of progression—although it was associated with *MET* amplification in only 10% of primary cancers, but in most cases of the metastatic tumors. This evidence suggested that *MET* amplification appears to give a selective advantage for the acquisition of metastatic phenotype and may be a late event in the progression of CRC [73].

Despite the increasing knowledge of the molecular bases of CRC progression, the exact mechanisms that trigger the metastatic spread are not fully understood. It has been recently observed that the pathways, which regulate the EMT, act as key mediators of the metastatic processes [74]. HGF/cMET axis may regulate the expression of *E*-cadherin, *N*-cadherin, and extracellular matrix degrading proteases, thus facilitating the invasiveness of tumor cells [76]. The metastasis-associated in colon cancer 1 (*MACC1*) gene has been recently described [77]. *MACC1* expression was reported to be markedly up-regulated in all stages of both primary CRC and distant metastases tissues, when compared with normal tissues. This biomarker may therefore represent an accurate prognostic indicator for the development of metastases. As the *MET* gene is a transcriptional target of *MACC1*, this latter may confer malignant potential and aggressiveness to CRC cells, thanks to its influence on the HGF/cMET pathway and on the MAPK axis [78]. In addition, MACC1 promotes proliferation, invasion and HGF-induced scattering of CRC cells *in vitro*, as well as tumor growth and metastases formation in mouse xenograft models [77].

## 6. *MET-*Targeting by MicroRNAs

MicroRNAs (miRNAs) are small non-coding RNAs of approximately 22–25 nucleotides that have emerged as critical regulators of gene expression by RNA interference. They are actively involved in many biological processes and play an important role in a number of diseases, including cancer [79]. In fact, each miRNA can target several genes and, in most cases, they are expressed in a tissue-specific manner. The expression of these molecules is deregulated in cancer cells compared with normal tissues, and experimental data showed that cancer phenotypes can be modified by targeting miRNA expression [80]. These findings have prompted researchers to develop miRNA-based anticancer therapies, which can consist in the blockade of miRNA expression in case of oncogenic miRNAs, or, on the other hand, in the replacement of malfunctioning or absent tumor suppressor miRNAs through the use of synthetic oligonucleotides (miRNA mimics) or virus-based constructs.

However, miRNA therapy still faces many issues, including tissue-specific delivery, poor cellular uptake of mimics, and potential off-target effects. In order to overcome these problems, efforts should be taken in developing new delivery methods or in maximizing target specificity.

Several miRNAs have been identified which target *MET* oncogene, including miR-34a, miR-199, miR-206, and miR-1 that could be challenged in therapies for silencing *MET*. We have recently observed that miR-1 is downregulated in CRC with respect to matched normal tissues and we have demonstrated that this miRNA can downregulate *MET* expression *in vitro* CRC models. In addition, re-expression of miR-1 in CRC cell lines leads to *MET*-driven reduction in cell proliferation and motility, thus suggesting that miR-1 can be a possible candidate for clinical trials of *MET* inhibitors in the treatment of metastatic CRC [70].

## 7. cMET as Prognostic Biomarker

Lee *et al.* [81] demonstrated that the expression patterns of RON and cMET, as assessed by IHC, were significantly associated with clinical outcome in patients with CRC. In particular, the results of their study showed that patients whose specimens presented high expression of cMET and RON had an 11-fold increased risk of tumor recurrence if compared to patients with specimens showing a low expression [81].

Since *MET* is associated with the progression and aggressiveness of CRC [68], several studies suggested a role for this gene as a prognostic biomarker, given also that an increased expression of cMET mRNA had already been observed in highly-metastatic cell lines [82]. Moreover, *in vivo* studies reported a correlation between cMET overexpression and TNM stage, and showed a significant increase in cMET according to tumor size and aggressiveness in primary CRC [75,83]. Takeuchi *et al.* [68] demonstrated that the overexpression of cMET increases according to the invasiveness of the primary CRC and to the presence of lymph node metastases [68]. A significant increment in the number of cMET mRNA copies was shown in early stages CRC (T1–T2), as compared with more advanced ones (T3–T4). In N1–N2 tumors, the mRNA copy number was also increased compared with N0 tumors (*p* < 0.03). These data suggest that cMET overexpression plays an important role in the development of loco-regional invasiveness in the early stages of CRC development [68]. Notably, these results are in line with the finding that higher serum preoperative levels of HGF are associated with poor survival in stage II-III CRC [32].

In CRC, the activation of HGF/cMET signaling pathway is not related to gene mutation but can occur either in a ligand-dependent manner or through a paracrine mechanism. Kammula *et al.* [69] demonstrated that the expression levels of both cMET and HGF mRNA significantly increased from the normal colonic mucosa to the matched primary tumors. Noteworthy, a multivariate model was designed to correlate the presence of HGF and/or cMET with outcome, showing that overexpression of cMET and/or HGF was independently associated with poorer overall survival.

Interestingly, in a study on resected CRC liver metastases, Osada *et al.* [84] measured the expression of cMET by Western blot analysis, as well as serum HGF levels. Results revealed that patients with high cMET expression in their tumors and high serum HGF levels had an increased risk of recurrence immediately after hepatectomy, resulting in an overall unfavorable prognosis [84].

Recently, *in vitro* studies showed that the irradiation of rectal cancer tumor cells induces an up-regulation of HGF expression and EMT. The subsequent production of cMET and the activation of its related pathway eventually boost the incidence of local and distant recurrence. In this regard, the inhibition of the HGF/cMET pathway may, therefore, represent a valid therapeutic approach for rectal cancer patients treated with preoperative chemoradiation, as it could potentially improve outcome by decreasing radiation-induced HGF up-regulation and metastatic potential [71].

In line with what has been previously done for breast cancer, a molecular classification has been recently proposed for CRC. Although a consensus has not been reached, Simon *et al.* [85] proposed a classification based on gene expression patterns. Data of 188 stage I-IV CRC patients were used to develop a molecular subtype classification, which was then validated in 543 stage II and III patients. The correlation with clinical characteristics and outcome was investigated and three major subtypes were identified (A, B, and C), based on biological features: deficiency in mismatch repair genes, epithelial proliferation, and EMT [85]. The subtype C was associated with the worst outcome, being characterized by an expression of mesenchymal genes—such as *MET*—and absence of benefit from adjuvant chemotherapy. On the other hand, a better clinical outcome was associated with A-type and B-type tumors, characterized by a more proliferative and epithelial phenotype, and expected benefit from adjuvant chemotherapy [85]. Another recent study investigated the association between c-Met expression and clinico-pathological characteristics on 590 CRC samples. *MET* overexpression was found in 17% of CRC tumors and was significantly associated with the gene expression of an EMT phenotype and a worse survival (HR 2.92; 95% CI: 1.45–5.92) [86].

## 8. cMET as Predictive Biomarker

Although anti-EGFR MoAbs cetuximab and panitumumab have established efficacy in advanced CRC, they achieve a response rate of only 10%–20% when used as a single agent in unselected chemo-refractory patients [87,88]. KRAS mutational status is the main predictor of primary resistance [89,90]. Similarly, mutations of other downstream effectors like NRAS, BRAF, or PIK3CA may affect response to cetuximab or panitumumab [91,92]. Indeed, the selection of patients with KRAS, NRAS, BRAF, and PIK3CA wild type tumors may achieve a response rate exceeding 40%. Unfortunately, patients who initially respond will eventually progress within three to 12 months, because of secondary resistance mechanisms [87,88].

Other mechanisms of resistance to anti-EGFR therapy may be represented by the activation of parallel pathways, such as HGF/cMET. It has been already established that *MET* amplification is a mechanism of acquired resistance to EGFR tyrosine kinase inhibitors (TKIs) in NSCLC harboring EGFR activating mutations [46,93]. Some emerging evidence suggest that amplification or overexpression of *MET* can play an important role for primary and secondary resistance to anti-EGFR therapy also in advanced CRC. In fact, in preclinical models, CRC cell lines with KRAS, BRAF, NRAS, and PIK3CA wild-type status developed resistance to cetuximab and panitumumab when ectopic cMET overexpression was induced by cDNA transfection. Experiments on xenografts derived from patients not exposed to anti-EGFR moAbs showed that treatment with cetuximab was not effective in mice engrafted with CRC specimens carrying *MET* amplification, suggesting that *MET* amplification may be involved in primary resistance [64]. Although rare, constitutional *MET* amplification characterizes a significant fraction of cetuximab-resistant cases that are wild type for KRAS, BRAF, NRAS, and PIK3CA. A previous retrospective study showed that *MET* overexpression in specimens obtained before starting treatment was associated with lack of benefit from cetuximab [94]. However, data from other series are conflicting, and given the low prevalence of *MET* amplification or overexpression in untreated metastatic CRC, its clinical validation as a predictive biomarker will require larger studies [95].

Recent data suggest that secondary KRAS mutations arising during treatment are responsible for about half cases of acquired resistance [96]. In the remaining cases, however, different mechanisms of resistance should be investigated. Bardelli and colleagues performed molecular analysis on tissue samples obtained before treatment with anti-EGFR moAbs and at the time of disease progression. In patients not developing KRAS mutations, the amplification and consequent overexpression of cMET was one of the most frequently mechanisms of acquired resistance observed. In some patients, rare *MET*-amplified cells may be found in tissue samples before treatment, suggesting that EGFR-targeted therapies may act as a selective pressure to expand a preexisting subclonal population of cancer cells with *MET* amplification in a Darwinian fashion [64].

Another mechanism for *MET*-induced resistance to anti-EGFR therapy is represented by its activation by the ligand—namely HGF. In preclinical models, stimulation with HGF is able to rescue cetuximab-sensitive CRC cell lines from EGFR inhibition in a dose-dependent manner through *MET*-induced activation of the AKT and MAPK pathways, even in absence of *MET* amplification [47]. These findings support the hypothesis that HGF overexpression by cancer cells themselves or by the surrounding stroma may be an independent mechanism of resistance to anti-EGFR therapy.

HGF/cMET axis seems to play an important role in primary and acquired resistance to anti-EGFR moAbs in advanced CRC. Above all, cMET is a druggable target and cMET inhibition represents a promising strategy to overcome resistance. In fact, the high prevalence of activated HGF/cMET pathway in human malignancies has driven a rapid growth of oncology drug-development programs, with several new agents targeting cMET in ongoing clinical trials [97]. These agents include the are summarized in Table 2 and described in the next sections:

### 8.1. Anti-HGF Monoclonal Antibodies

AMG 102 (Rilotumumab, Amgen, Thousand Oaks, CA, USA) is a humanized monoclonal antibody directed against HGF, which blocks the interaction between HGF and cMET [98]. A randomized phase Ib/II trial of panitumumab in combination with rilotumumab or placebo was carried out for patients with KRAS wild-type, advanced CRC [99]. The recommended phase II doses were the standard dose of panitumumab and 10 mg/kg of rilotumumab biweekly. A total of 142 patients were included in the expansion phase II cohort of the study after failure of prior first line irinotecan- and/or oxaliplatin-based regimens. The response rate—namely the study primary endpoint—was 31% of patients in the combination arm *vs.* 21% in the panitumumab plus placebo arm. The median duration of response with the experimental arm was 5.1 months compared with 3.7 months for panitumumab alone. Median PFS showed a trend for improvement in the combination treatment at 5.2 months compared with 3.7 months in the panitumumab-placebo arm [100]. Expression of cMET was measured by IHC on archival tumor samples, which were scored for cMET expression with a staining intensity between 0 and 3; to determine the percentage of tumor cells expressing cMET in a sample, tumor cells with a staining intensity of at least 1 were considered positive—with positive tumor cells >50% considered as high cMET expression. However, the statistical interaction between cMET expression and treatment arm was not significant—probably due to small sample size and the method of IHC assessment. Currently, there is no development program of rilotumumab in advanced CRC, although a better selection of cMET positive tumors and the evaluation of rilotumumab as single agent in both KRAS wild-type and mutated tumors could improve the therapeutic index of this agent. Moreover, this agent continues to be evaluated in other malignancies, reaching phase III development in gastric cancer.

AV-299 (Ficlatuzumab, AVEO, Cambridge, MA, USA) is a monoclonal antibody directed against HGF and is currently being developed in NSCLC [101].

### 8.2. Anti-MET Monoclonal Antibodies

MetMAb (Onartuzumab, Genentech, San Francisco, CA, USA) is a monovalent monoclonal antibody directed against cMET, which prevents HGF from binding to the cMET receptor and, ultimately, blocks ligand-induced cMET dimerization and intracellular domain activation. Of note, this drug was developed as a single-armed humanized modified anti-*MET* antibody, which acts as a monovalent antibody to avoid agonistic activity that may occur when a bivalent antibody binds to two separate cMET molecules [102]. In CRC, a phase II randomized study of onartuzumab *versus* placebo, both in association with the standard of fist-line treatment (modified FOLFOX-6 plus bevacizumab) was recently closed to enrollment with the primary endpoint of detecting progression-free survival differences [103]. The evaluation of cMET is a pre-specified retrospective analysis that will be carried out using the same criteria described by Spiegel *et al.* [104] for NSCLC. Briefly, intensity of *MET* staining on tumor cells will be scored in four categories: negative, weak, moderate, and strong. *MET* positivity will be defined as the majority (≥50%) of tumor cells with a moderate or strong staining intensity. Currently, the final results of the phase II CRC study are pending.

### 8.3. Tyrosine Kinase Inhibitors

TKIs are small molecules, which compete for the adenosine triphosphate binding site in the tyrosine kinase domain of cMET, thus preventing receptor transactivation and recruitment of downstream effectors. They can be classified as unselective or selective inhibitors.

Among unselective TKIs, crizotinib (PF-02341066; Pfizer, New York, NY, USA) is an orally available 2-amino-3-benzyloxy-5-arylpyridine compound, initially developed to target cMET. After the discovery of the anaplastic lymphoma kinase (ALK), it was renamed as the ALK-targeted therapy, which was approved by the US Food and Drug Administration for patients with NSCLC harboring EML4-ALK gene rearrangement [105].

Foretinib (XL 880, EXEL 2880, GSK 1363089; Exelixis/GlaxoSmithKline, Philadelphia, PA, USA) is an oral multikinase inhibitor developed to target cMET and several other tyrosine kinases involved in angiogenesis. It is an ATP-competitive inhibitor and binds to the ATP pocket of cMET and VEGFR-2 tyrosine kinase domains (dual VEFGR-2/cMET inhibitor) [106].

Cabozantinib (XL-184/BMS-907351; Exelixis, South San Francisco, CA, USA/Bristol-Myers Squibb, Princeton, NJ, USA) is an orally administrated TKI, which targets cMET and VEGFR1-3. It can pass the blood-brain barrier and presents a marked activity in blastic bone metastases deriving from prostate cancer [107].

However, unselective cMET inhibitors such as foretinib amd cabozantinib are not currently being evaluated in patients with advanced CRC.

Tivantinib (ARQ-197, ArQule (Woburn, MA, USA), in partnership with Daiichi Sankyo (Tokyo, Japan) and Asian licensee Kyowa Hakko Kirin (Tokyo, Japan)) is a highly-selective, oral, non-adenosine triphosphate (ATP)-competitive cMET inhibitor. A phase I/II randomized study of biweekly schedule of irinotecan and cetuximab associated with tivantinib or placebo was conducted after failure of first line therapy for patients with KRAS wild-type advanced CRC [108]. At the recommended phase II dose of tivantinib (360 mg twice daily), the investigational drug failed to show any statistically significant improvement of the primary endpoint—progression-free survival—as median time was 8.3 months in tivantinib arm *vs.* 7.3 months in the placebo arm (hazard ratio 0.85, 95% confidence interval, 0.55–1.33; *p* = 0.38) [109]. There appeared to be some benefit for PFS in the subgroup that received previous first-line oxaliplatin-based treatment. However, this advantage was obtained from a retrospective subgroup analysis and was not statistically significant (hazard ratio 0.66 with a confidence interval of 0.41–1.09).

Finally, INCB028060, PF-04217903, E7050, JNJ-38877605, and BMS-777607 are potent and selective inhibitors of cMet receptor tyrosine kinase and completed the first in human studies.

## 9. Conclusions

*MET* is considered a promising prognostic biomarker in early stage CRC. In advanced disease, the potential predictive role of *MET* for benefit from molecularly targeted agents will be explored, and hopefully confirmed, by prospective studies. The optimal methods of assessment of cMET as biomarkers are still being developed. As a result, patient selection in current and prior studies has been underemphasized. For future analyses, a strong recommendation for a select patient population should be encouraged based on biomarker data, since this is more likely to be representative of tumor biology and the therapeutic potential of targeted therapies. Thus far, most studies have assessed the HGF/cMET axis in tumors at baseline. However, HGF/cMET activation has been implicated as an important mechanism of metastatic progression and treatment resistance. Future studies should address the need to biopsy the most recent site of metastatic progression, since targeted therapy directed against HGF/cMET may be of considerable value in surmounting both primary and acquired resistance in selected CRC populations [110].

In this regard, *MET* could be a “mixed” biomarker—with negative prognostic value and positive predictive effect. However, the potential application of *MET* routine assessment in the clinical practice will strongly be dependent on the possibility to conduct adequately powered, biomarkers-driven clinical trials with the goal of personalized medicine.

## Figures and Tables

**Table 1:** Cellular and biological processes related to the HGF/cMET pathway.

Cellular/biological process	HGF/cMET pathway involvement
*Embryogenesis*	EMT of myogenic progenitor cells and development of muscular tissue Development of neuronal precursors, liver and placental tissue Regeneration after injury in different epithelial tissues
*Tissue regeneration*	Wound repair of the skin Induction of DNA synthesis and liver regeneration
*Cell proliferation and survival*	Activation of cell proliferation, survival and migration
*Cytoskeleton*	Involvement in cell adhesion, actin reorganization and cell growth
*Scattering and cell motility*	Induction of cell motility, invasion and metastatization

Abbreviations: EMT, epithelial-mesenchymal transition; HGF, hepatocyte growth factor; *MET*, mesenchymal-epithelial transition.

**Table 2:** Mesenchymal-epithelial transition (*MET*) inhibitors evaluated in clinical trials.

Company	Compound	Mechanism of action	Clinical development
Amgen	Rilotumumab	HGF IgG2 Mab	Phase II:CRC
Aveo	Ficlatuzumab	HGF IgG1 Mab	Phase II: NSCLC
Genetech/Roche	Onartuzumab	*MET* IgG1 Mab	Phase II: NSCLC Phase II: CRC
Pfizer	Crizotinib	*MET* TKI Other TKI inhibition: ALK, RON, AXL, TIE2	Phase IV: NSCLC
GlaxoSmithKline	Foretinib	*MET* TKI Other TKI inhibition: VEGFR2, AXL, PDGFR, KIT, FLT3, TIE2	Phase II:
Exelis	Cabozantinib	*MET* TKI Other TKI inhibition: VEGFR2, RET, KIT, FLT3, TIE2	Phase II: NSCLC
ArQule	Tivantinib	*MET* TKI	Phase II:CRC

Abbreviations: CRC, colon rectal cancer; NSCLC, non-small-cell lung cancer; RON, macrophage-stimulating protein receptor; PDGFR, platelet-derived growth factor receptor; TIE2, angiopoietin 1 receptor.
